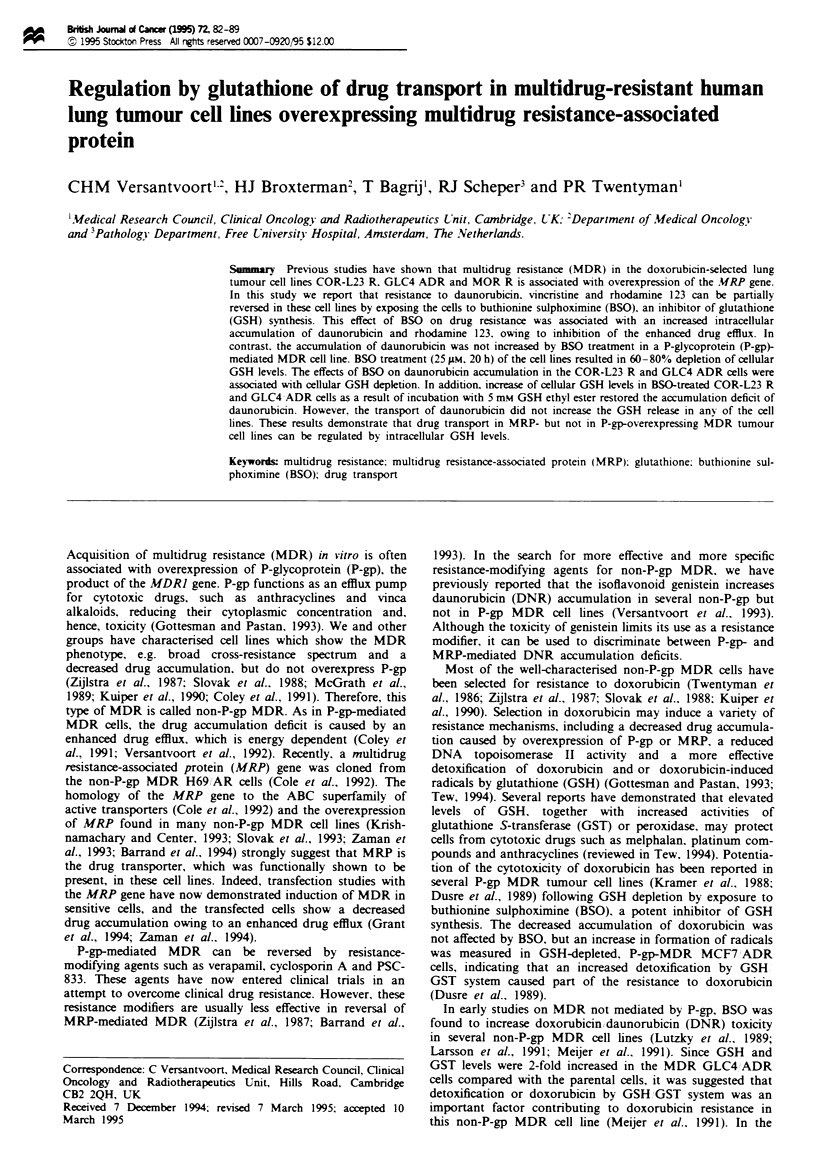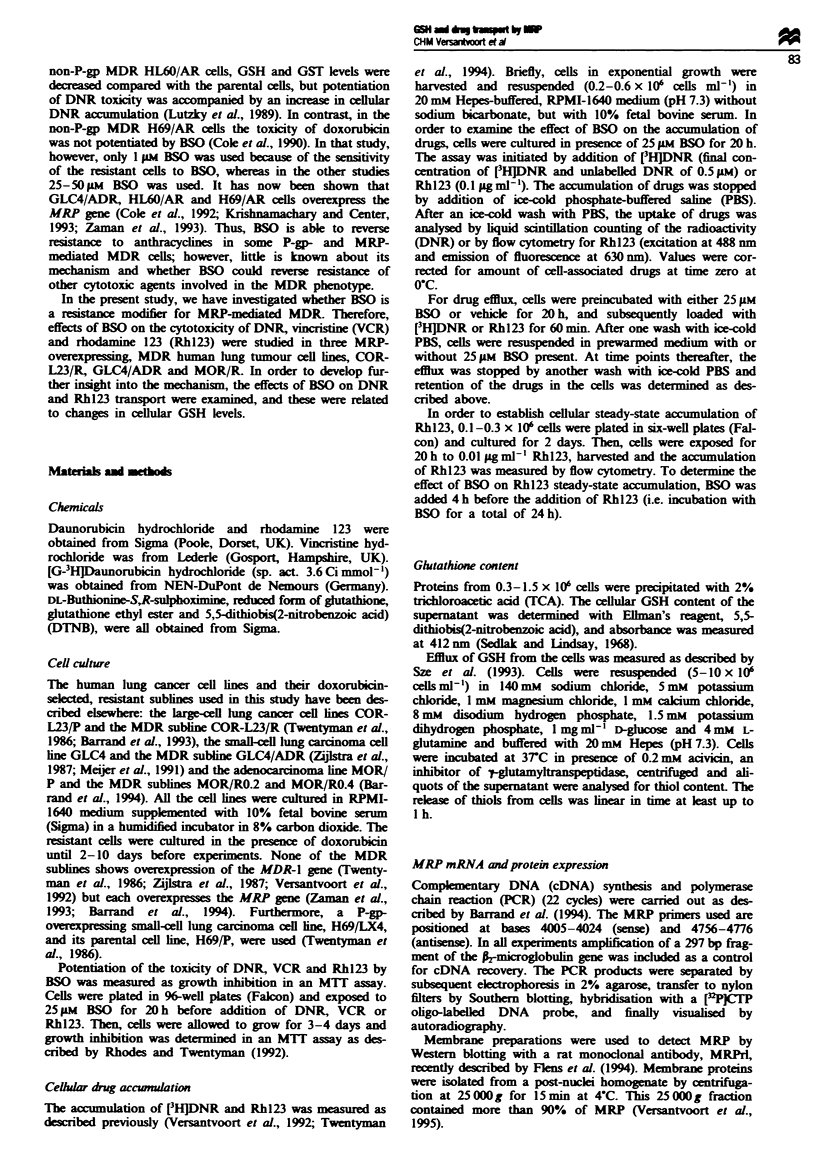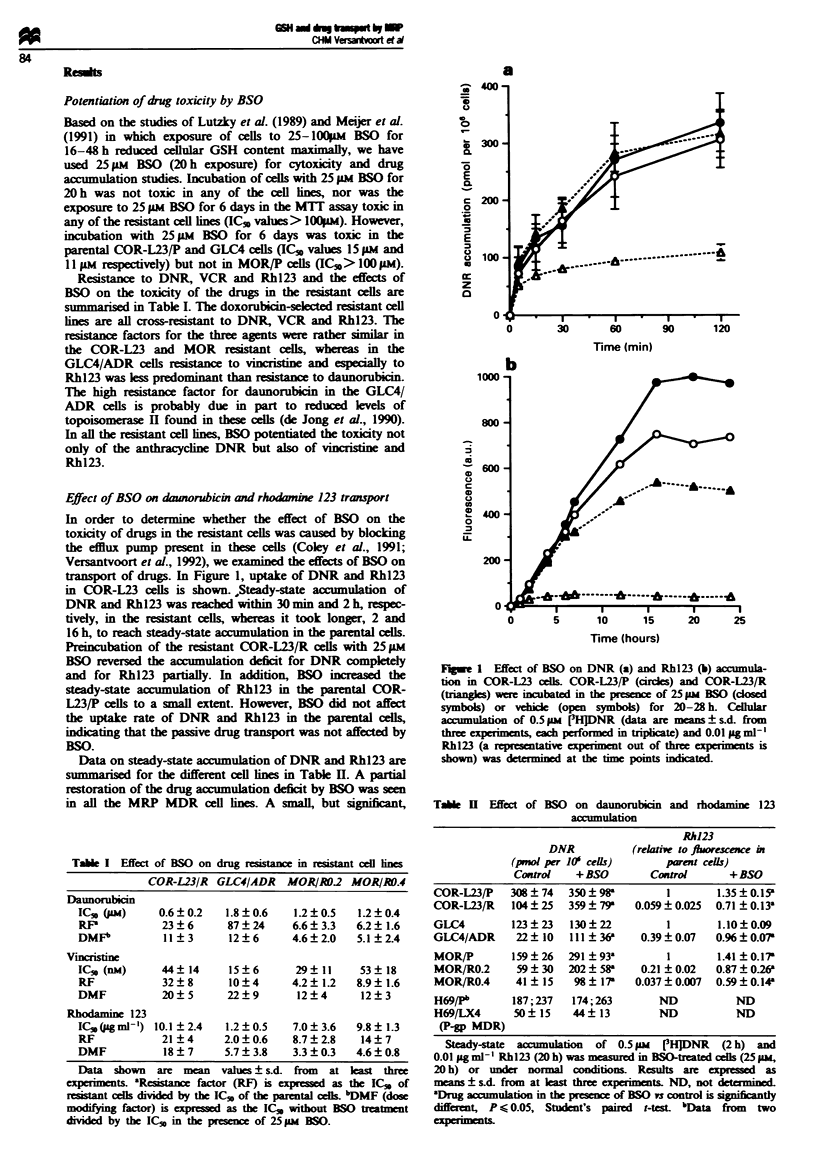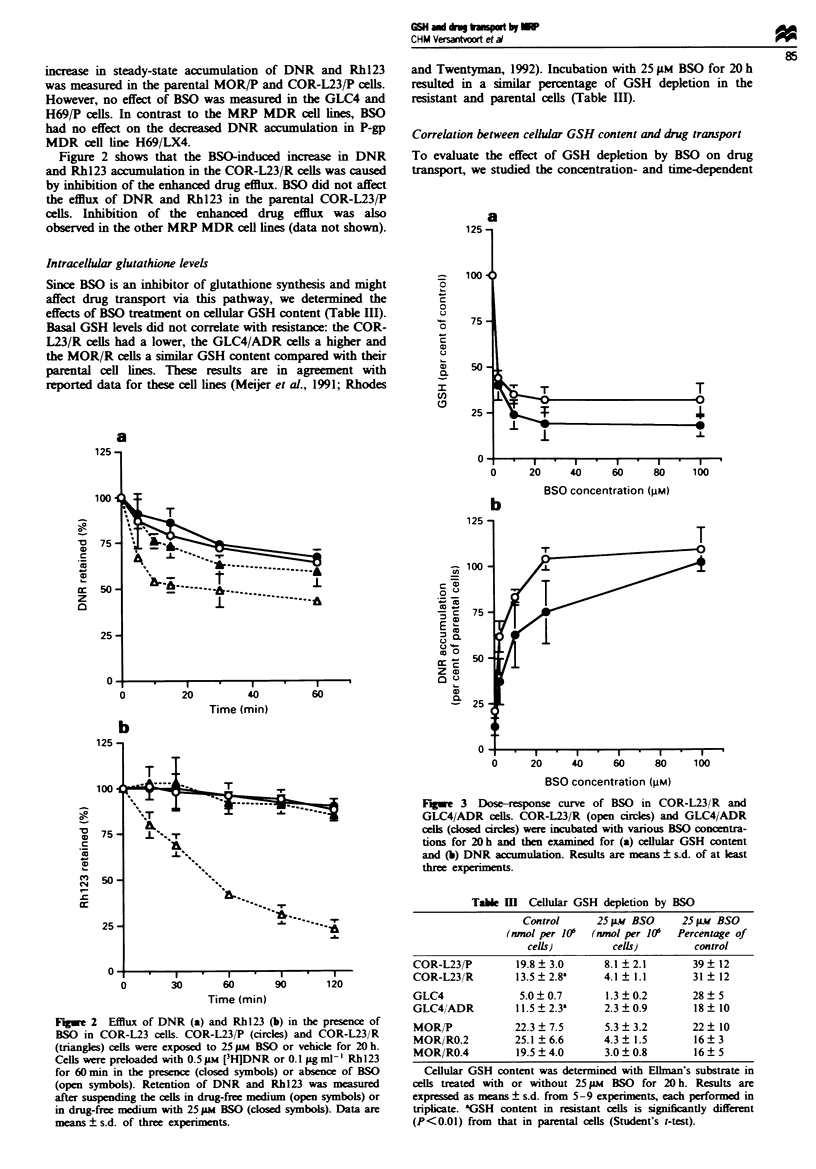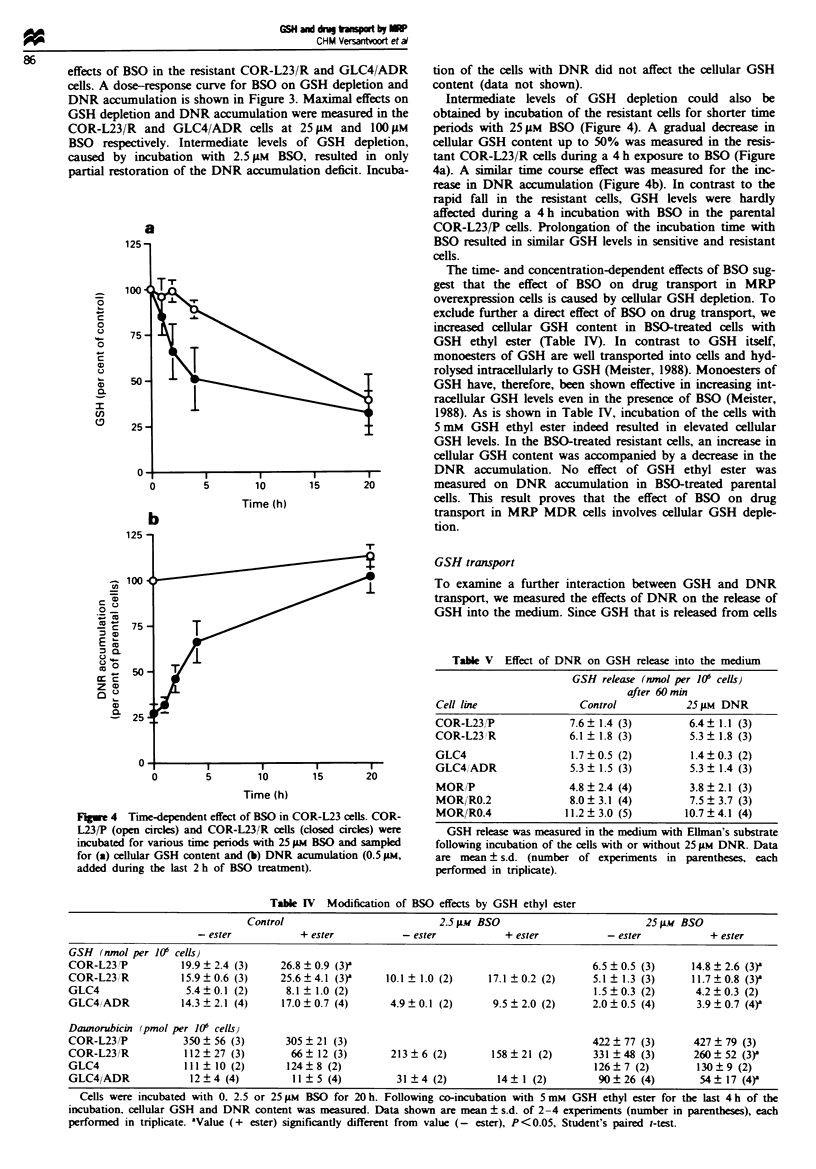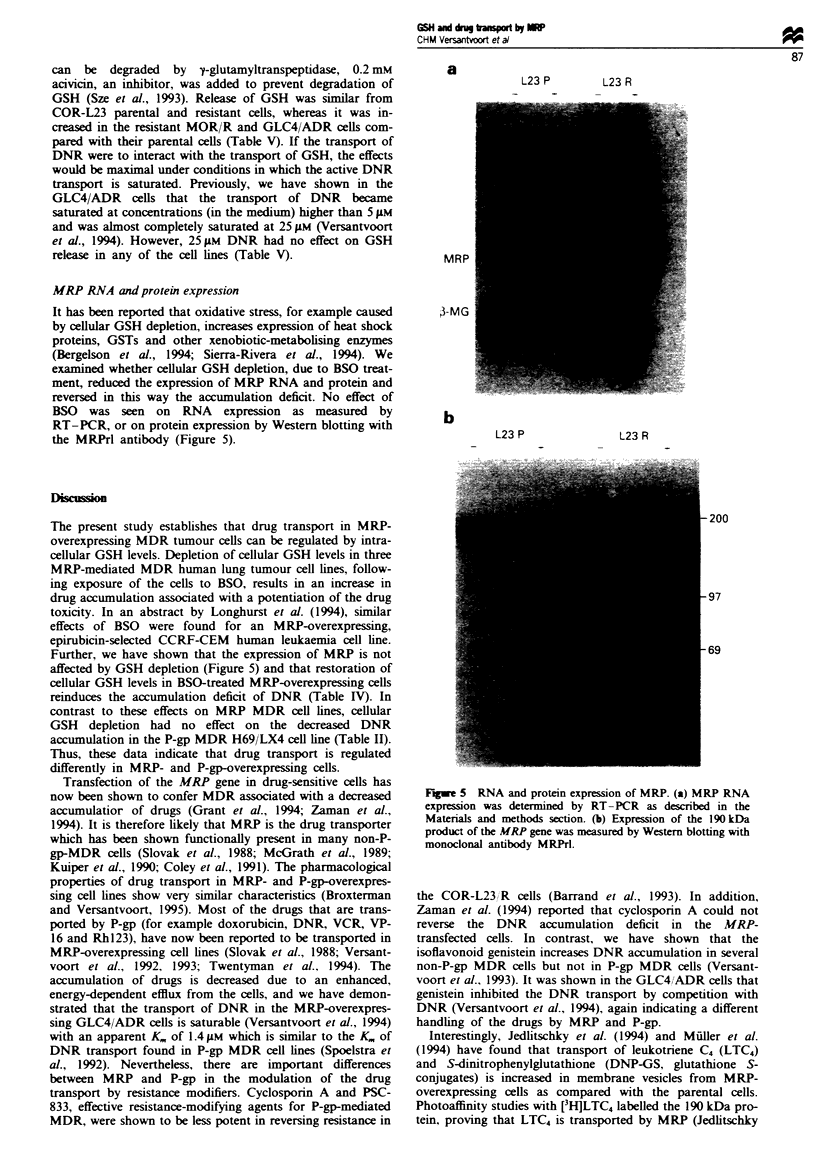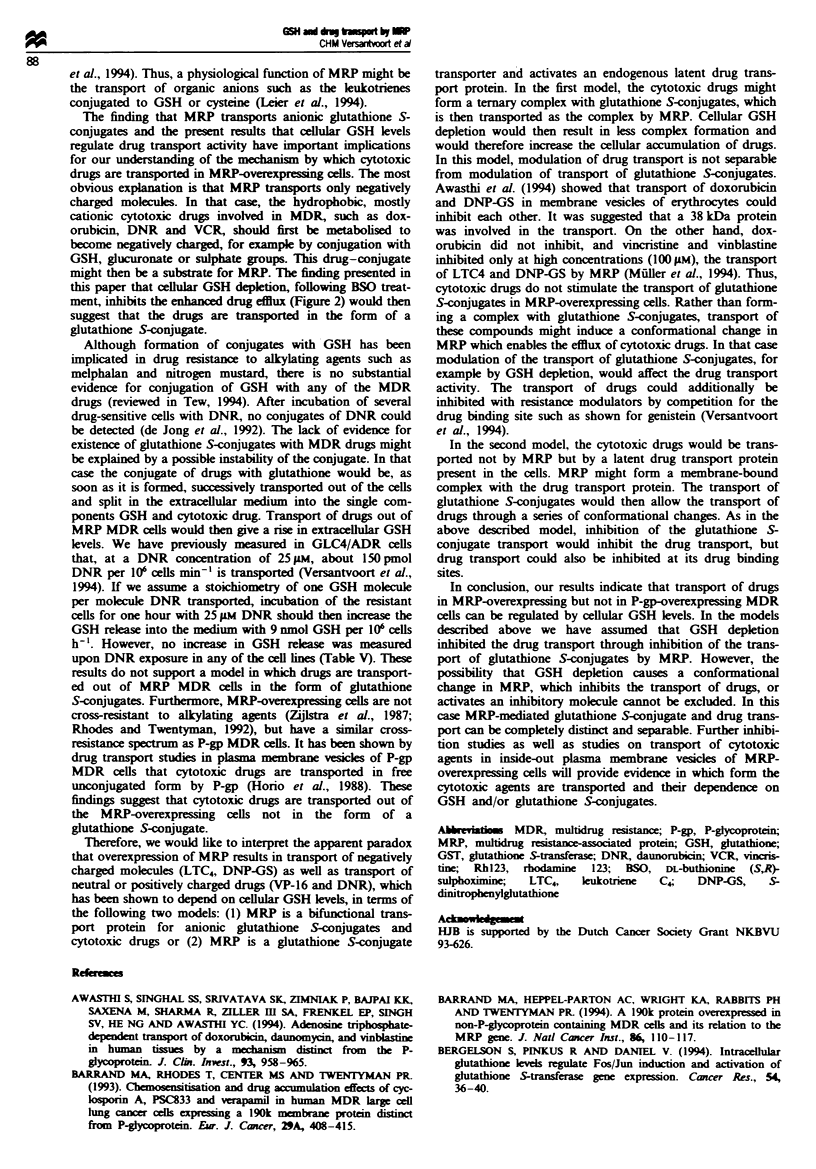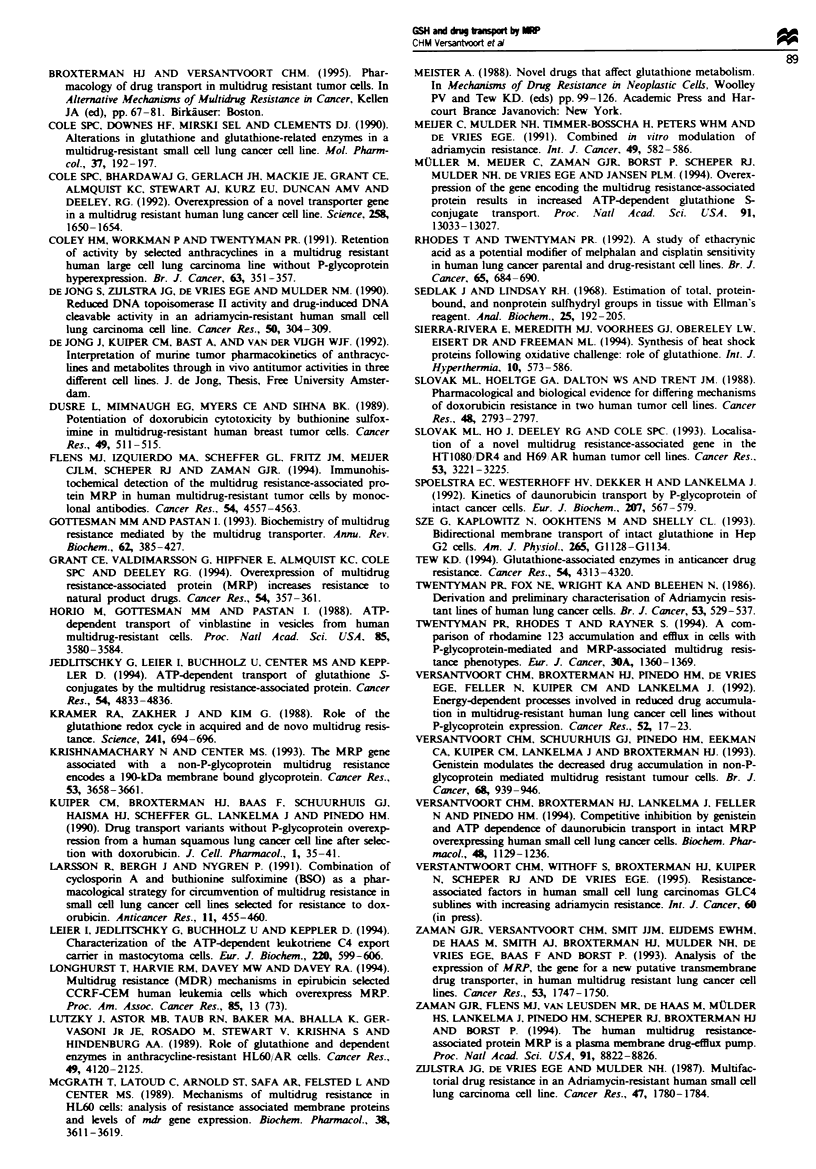# Regulation by glutathione of drug transport in multidrug-resistant human lung tumour cell lines overexpressing multidrug resistance-associated protein.

**DOI:** 10.1038/bjc.1995.281

**Published:** 1995-07

**Authors:** C. H. Versantvoort, H. J. Broxterman, T. Bagrij, R. J. Scheper, P. R. Twentyman

**Affiliations:** Medical Research Council, Clinical Oncology and Radiotherapeutics Unit, Cambridge, UK.

## Abstract

**Images:**


British Journal of Cancer (1995) 72, 82-89

© 1995 Stockton Press All rights reserved 0007-0920/95 $12.00

### Regulation by glutathione of drug transport in multidrug-resistant human lung tumour cell lines overexpressing multidrug resistance-associated protein

CHM Versantvoort[1,2], HJ Broxterman[2], T Bagrij[1], RJ Scheper[3] and PR Twentyman[1]

[1]*Medical Research Council, Clinical Oncology and Radiotherapeutics Unit, Cambridge, UK;* [2]*Department of Medical Oncology and* [3]*Pathology Department, Free University Hospital, Amsterdam, The Netherlands.*

**Summary** Previous studies have shown that multidrug resistance (MDR) in the doxorubicin-selected lung tumour cell lines COR-L23 R, GLC4 ADR and MOR R is associated with overexpression of the *MRP* gene. In this study we report that resistance to daunorubicin, vincristine and rhodamine 123 can be partially reversed in these cell lines by exposing the cells to buthionine sulphoximine (BSO), an inhibitor of glutathione (GSH) synthesis. This effect of BSO on drug resistance was associated with an increased intracellular accumulation of daunorubicin and rhodamine 123, owing to inhibition of the enhanced drug efflux. In contrast, the accumulation of daunorubicin was not increased by BSO treatment in a P-glycoprotein (P-gp)-mediated MDR cell line. BSO treatment (25 μM, 20 h) of the cell lines resulted in 60-80% depletion of cellular GSH levels. The effects of BSO on daunorubicin accumulation in the COR-L23 R and GLC4 ADR cells were associated with cellular GSH depletion. In addition, increase of cellular GSH levels in BSO-treated COR-L23 R and GLC4 ADR cells as a result of incubation with 5 mM GSH ethyl ester restored the accumulation deficit of daunorubicin. However, the transport of daunorubicin did not increase the GSH release in any of the cell lines. These results demonstrate that drug transport in MRP- but not in P-gp-overexpressing MDR tumour cell lines can be regulated by intracellular GSH levels.

**Keywords:** multidrug resistance; multidrug resistance-associated protein (MRP); glutathione; buthionine sulphoximine (BSO); drug transport

Acquisition of multidrug resistance (MDR) *in vitro* is often associated with overexpression of P-glycoprotein (P-gp), the product of the *MDR1* gene. P-gp functions as an efflux pump for cytotoxic drugs, such as anthracyclines and vinca alkaloids, reducing their cytoplasmic concentration and, hence, toxicity (Gottesman and Pastan, 1993). We and other groups have characterised cell lines which show the MDR phenotype, e.g. broad cross-resistance spectrum and a decreased drug accumulation, but do not overexpress P-gp (Zijlstra *et al.*, 1987; Slovak *et al.*, 1988; McGrath *et al.*, 1989; Kuiper *et al.*, 1990; Coley *et al.*, 1991). Therefore, this type of MDR is called non-P-gp MDR. As in P-gp-mediated MDR cells, the drug accumulation deficit is caused by an enhanced drug efflux, which is energy dependent (Coley *et al.*, 1991; Versantvoort *et al.*, 1992). Recently, a *m*ultidrug *r*esistance-associated *p*rotein (*MRP*) gene was cloned from the non-P-gp MDR H69/AR cells (Cole *et al.*, 1992). The homology of the *MRP* gene to the ABC superfamily of active transporters (Cole *et al.*, 1992) and the overexpression of *MRP* found in many non-P-gp MDR cell lines (Krishnamachary and Center, 1993; Slovak *et al.*, 1993; Zaman *et al.*, 1993; Barrand *et al.*, 1994) strongly suggest that MRP is the drug transporter, which was functionally shown to be present, in these cell lines. Indeed, transfection studies with the *MRP* gene have now demonstrated induction of MDR in sensitive cells, and the transfected cells show a decreased drug accumulation owing to an enhanced drug efflux (Grant *et al.*, 1994; Zaman *et al.*, 1994).

P-gp-mediated MDR can be reversed by resistance-modifying agents such as verapamil, cyclosporin A and PSC-833. These agents have now entered clinical trials in an attempt to overcome clinical drug resistance. However, these resistance modifiers are usually less effective in reversal of MRP-mediated MDR (Zijlstra *et al.*, 1987; Barrand *et al.*, 1993). In the search for more effective and more specific resistance-modifying agents for non-P-gp MDR, we have previously reported that the isoflavonoid genistein increases daunorubicin (DNR) accumulation in several non-P-gp but not in P-gp MDR cell lines (Versantvoort *et al.*, 1993). Although the toxicity of genistein limits its use as a resistance modifier, it can be used to discriminate between P-gp- and MRP-mediated DNR accumulation deficits.

Most of the well-characterised non-P-gp MDR cells have been selected for resistance to doxorubicin (Twentyman *et al.*, 1986; Zijlstra *et al.*, 1987; Slovak *et al.*, 1988; Kuiper *et al.*, 1990). Selection in doxorubicin may induce a variety of resistance mechanisms, including a decreased drug accumulation caused by overexpression of P-gp or MRP, a reduced DNA topoisomerase II activity and a more effective detoxification of doxorubicin and/or doxorubicin-induced radicals by glutathione (GSH) (Gottesman and Pastan, 1993; Tew, 1994). Several reports have demonstrated that elevated levels of GSH, together with increased activities of glutathione *S*-transferase (GST) or peroxidase, may protect cells from cytotoxic drugs such as melphalan, platinum compounds and anthracyclines (reviewed in Tew, 1994). Potentiation of the cytotoxicity of doxorubicin has been reported in several P-gp MDR tumour cell lines (Kramer *et al.*, 1988; Dusre *et al.*, 1989) following GSH depletion by exposure to buthionine sulphoximine (BSO), a potent inhibitor of GSH synthesis. The decreased accumulation of doxorubicin was not affected by BSO, but an increase in formation of radicals was measured in GSH-depleted, P-gp-MDR MCF7/ADR cells, indicating that an increased detoxification by GSH/GST system caused part of the resistance to doxorubicin (Dusre *et al.*, 1989).

In early studies on MDR not mediated by P-gp, BSO was found to increase doxorubicin/daunorubicin (DNR) toxicity in several non-P-gp MDR cell lines (Lutzky *et al.*, 1989; Larsson *et al.*, 1991; Meijer *et al.*, 1991). Since GSH and GST levels were 2-fold increased in the MDR GLC4/ADR cells compared with the parental cells, it was suggested that detoxification or doxorubicin by GSH/GST system was an important factor contributing to doxorubicin resistance in this non-P-gp MDR cell line (Meijer *et al.*, 1991). In the

Correspondence: C Versantvoort, Medical Research Council, Clinical Oncology and Radiotherapeutics Unit, Hills Road, Cambridge CB2 2QH, UK
Received 7 December 1994; revised 7 March 1995; accepted 10 March 1995

non-P-gp MDR HL60/AR cells, GSH and GST levels were decreased compared with the parental cells, but potentiation of DNR toxicity was accompanied by an increase in cellular DNR accumulation (Lutzky *et al.*, 1989). In contrast, in the non-P-gp MDR H69/AR cells the toxicity of doxorubicin was not potentiated by BSO (Cole *et al.*, 1990). In that study, however, only 1 μM BSO was used because of the sensitivity of the resistant cells to BSO, whereas in the other studies 25–50 μM BSO was used. It has now been shown that GLC4/ADR, HL60/AR and H69/AR cells overexpress the *MRP* gene (Cole *et al.*, 1992; Krishnamachary and Center, 1993; Zaman *et al.*, 1993). Thus, BSO is able to reverse resistance to anthracyclines in some P-gp- and MRP-mediated MDR cells; however, little is known about its mechanism and whether BSO could reverse resistance of other cytotoxic agents involved in the MDR phenotype.

In the present study, we have investigated whether BSO is a resistance modifier for MRP-mediated MDR. Therefore, effects of BSO on the cytotoxicity of DNR, vincristine (VCR) and rhodamine 123 (Rh123) were studied in three MRP-overexpressing, MDR human lung tumour cell lines, COR-L23/R, GLC4/ADR and MOR/R. In order to develop further insight into the mechanism, the effects of BSO on DNR and Rh123 transport were examined, and these were related to changes in cellular GSH levels.

### Materials and methods

### *Chemicals*

Daunorubicin hydrochloride and rhodamine 123 were obtained from Sigma (Poole, Dorset, UK). Vincristine hydrochloride was from Lederle (Gosport, Hampshire, UK). [G-$^3$H]Daunorubicin hydrochloride (sp. act. 3.6 Ci mmol$^{-1}$) was obtained from NEN-DuPont de Nemours (Germany). DL-Buthionine-*S,R*-sulphoximine, reduced form of glutathione, glutathione ethyl ester and 5,5-dithiobis(2-nitrobenzoic acid) (DTNB), were all obtained from Sigma.

### *Cell culture*

The human lung cancer cell lines and their doxorubicin-selected, resistant sublines used in this study have been described elsewhere: the large-cell lung cancer cell lines COR-L23/P and the MDR subline COR-L23/R (Twentyman *et al.*, 1986; Barrand *et al.*, 1993), the small-cell lung carcinoma cell line GLC4 and the MDR subline GLC4/ADR (Zijlstra *et al.*, 1987; Meijer *et al.*, 1991) and the adenocarcinoma line MOR/P and the MDR sublines MOR/R0.2 and MOR/R0.4 (Barrand *et al.*, 1994). All the cell lines were cultured in RPMI-1640 medium supplemented with 10% fetal bovine serum (Sigma) in a humidified incubator in 8% carbon dioxide. The resistant cells were cultured in the presence of doxorubicin until 2–10 days before experiments. None of the MDR sublines shows overexpression of the *MDR*-1 gene (Twentyman *et al.*, 1986; Zijlstra *et al.*, 1987; Versantvoort *et al.*, 1992) but each overexpresses the *MRP* gene (Zaman *et al.*, 1993; Barrand *et al.*, 1994). Furthermore, a P-gp-overexpressing small-cell lung carcinoma cell line, H69/LX4, and its parental cell line, H69/P, were used (Twentyman *et al.*, 1986).

Potentiation of the toxicity of DNR, VCR and Rh123 by BSO was measured as growth inhibition in an MTT assay. Cells were plated in 96-well plates (Falcon) and exposed to 25 μM BSO for 20 h before addition of DNR, VCR or Rh123. Then, cells were allowed to grow for 3–4 days and growth inhibition was determined in an MTT assay as described by Rhodes and Twentyman (1992).

### *Cellular drug accumulation*

The accumulation of [$^3$H]DNR and Rh123 was measured as described previously (Versantvoort *et al.*, 1992; Twentyman *et al.*, 1994). Briefly, cells in exponential growth were harvested and resuspended ($0.2-0.6 \times 10^6$ cells ml$^{-1}$) in 20 mM Hepes-buffered, RPMI-1640 medium (pH 7.3) without sodium bicarbonate, but with 10% fetal bovine serum. In order to examine the effect of BSO on the accumulation of drugs, cells were cultured in presence of 25 μM BSO for 20 h. The assay was initiated by addition of [$^3$H]DNR (final concentration of [$^3$H]DNR and unlabelled DNR of 0.5 μM) or Rh123 (0.1 μg ml$^{-1}$). The accumulation of drugs was stopped by addition of ice-cold phosphate-buffered saline (PBS). After an ice-cold wash with PBS, the uptake of drugs was analysed by liquid scintillation counting of the radioactivity (DNR) or by flow cytometry for Rh123 (excitation at 488 nm and emission of fluorescence at 630 nm). Values were corrected for amount of cell-associated drugs at time zero at 0°C.

For drug efflux, cells were preincubated with either 25 μM BSO or vehicle for 20 h, and subsequently loaded with [$^3$H]DNR or Rh123 for 60 min. After one wash with ice-cold PBS, cells were resuspended in prewarmed medium with or without 25 μM BSO present. At time points thereafter, the efflux was stopped by another wash with ice-cold PBS and retention of the drugs in the cells was determined as described above.

In order to establish cellular steady-state accumulation of Rh123, $0.1-0.3 \times 10^6$ cells were plated in six-well plates (Falcon) and cultured for 2 days. Then, cells were exposed for 20 h to 0.01 μg ml$^{-1}$ Rh123, harvested and the accumulation of Rh123 was measured by flow cytometry. To determine the effect of BSO on Rh123 steady-state accumulation, BSO was added 4 h before the addition of Rh123 (i.e. incubation with BSO for a total of 24 h).

### *Glutathione content*

Proteins from $0.3-1.5 \times 10^6$ cells were precipitated with 2% trichloroacetic acid (TCA). The cellular GSH content of the supernatant was determined with Ellman's reagent, 5,5-dithiobis(2-nitrobenzoic acid), and absorbance was measured at 412 nm (Sedlak and Lindsay, 1968).

Efflux of GSH from the cells was measured as described by Sze *et al.* (1993). Cells were resuspended ($5-10 \times 10^6$ cells ml$^{-1}$) in 140 mM sodium chloride, 5 mM potassium chloride, 1 mM magnesium chloride, 1 mM calcium chloride, 8 mM disodium hydrogen phosphate, 1.5 mM potassium dihydrogen phosphate, 1 mg ml$^{-1}$ D-glucose and 4 mM L-glutamine and buffered with 20 mM Hepes (pH 7.3). Cells were incubated at 37°C in presence of 0.2 mM acivicin, an inhibitor of γ-glutamyltranspeptidase, centrifuged and aliquots of the supernatant were analysed for thiol content. The release of thiols from cells was linear in time at least up to 1 h.

### *MRP mRNA and protein expression*

Complementary DNA (cDNA) synthesis and polymerase chain reaction (PCR) (22 cycles) were carried out as described by Barrand *et al.* (1994). The MRP primers used are positioned at bases 4005–4024 (sense) and 4756–4776 (antisense). In all experiments amplification of a 297 bp fragment of the $\beta_2$-microglobulin gene was included as a control for cDNA recovery. The PCR products were separated by subsequent electrophoresis in 2% agarose, transfer to nylon filters by Southern blotting, hybridisation with a [$^{32}$P]CTP oligo-labelled DNA probe, and finally visualised by autoradiography.

Membrane preparations were used to detect MRP by Western blotting with a rat monoclonal antibody, MRPrl, recently described by Flens *et al.* (1994). Membrane proteins were isolated from a post-nuclei homogenate by centrifugation at 25 000 *g* for 15 min at 4°C. This 25 000 *g* fraction contained more than 90% of MRP (Versantvoort *et al.*, 1995).

### Results

### *Potentiation of drug toxicity by BSO*

Based on the studies of Lutzky *et al.* (1989) and Meijer *et al.* (1991) in which exposure of cells to 25–100μM BSO for 16–48 h reduced cellular GSH content maximally, we have used 25 μM BSO (20 h exposure) for cytoxicity and drug accumulation studies. Incubation of cells with 25 μM BSO for 20 h was not toxic in any of the cell lines, nor was the exposure to 25 μM BSO for 6 days in the MTT assay toxic in any of the resistant cell lines ($IC_{50}$ values > 100μM). However, incubation with 25 μM BSO for 6 days was toxic in the parental COR-L23/P and GLC4 cells ($IC_{50}$ values 15 μM and 11 μM respectively) but not in MOR/P cells ($IC_{50}$ > 100 μM).

Resistance to DNR, VCR and Rh123 and the effects of BSO on the toxicity of the drugs in the resistant cells are summarised in Table I. The doxorubicin-selected resistant cell lines are all cross-resistant to DNR, VCR and Rh123. The resistance factors for the three agents were rather similar in the COR-L23 and MOR resistant cells, whereas in the GLC4/ADR cells resistance to vincristine and especially to Rh123 was less predominant than resistance to daunorubicin. The high resistance factor for daunorubicin in the GLC4/ADR cells is probably due in part to reduced levels of topoisomerase II found in these cells (de Jong *et al.*, 1990). In all the resistant cell lines, BSO potentiated the toxicity not only of the anthracycline DNR but also of vincristine and Rh123.

### *Effect of BSO on daunorubicin and rhodamine 123 transport*

In order to determine whether the effect of BSO on the toxicity of drugs in the resistant cells was caused by blocking the efflux pump present in these cells (Coley *et al.*, 1991; Versantvoort *et al.*, 1992), we examined the effects of BSO on transport of drugs. In Figure 1, uptake of DNR and Rh123 in COR-L23 cells is shown. Steady-state accumulation of DNR and Rh123 was reached within 30 min and 2 h, respectively, in the resistant cells, whereas it took longer, 2 and 16 h, to reach steady-state accumulation in the parental cells. Preincubation of the resistant COR-L23/R cells with 25 μM BSO reversed the accumulation deficit for DNR completely and for Rh123 partially. In addition, BSO increased the steady-state accumulation of Rh123 in the parental COR-L23/P cells to a small extent. However, BSO did not affect the uptake rate of DNR and Rh123 in the parental cells, indicating that the passive drug transport was not affected by BSO.

Data on steady-state accumulation of DNR and Rh123 are summarised for the different cell lines in Table II. A partial restoration of the drug accumulation deficit by BSO was seen in all the MRP MDR cell lines. A small, but significant,

Table I Effect of BSO on drug resistance in resistant cell lines

| | COR-L23/R | GLC4/ADR | MOR/R0.2 | MOR/R0.4 |
|---|---|---|---|---|
| Daunorubicin | | | | |
| $IC_{50}$ (μM) | 0.6 ± 0.2 | 1.8 ± 0.6 | 1.2 ± 0.5 | 1.2 ± 0.4 |
| RF[a] | 23 ± 6 | 87 ± 24 | 6.6 ± 3.3 | 6.2 ± 1.6 |
| DMF[b] | 11 ± 3 | 12 ± 6 | 4.6 ± 2.0 | 5.1 ± 2.4 |
| Vincristine | | | | |
| $IC_{50}$ (nM) | 44 ± 14 | 15 ± 6 | 29 ± 11 | 53 ± 18 |
| RF | 32 ± 8 | 10 ± 4 | 4.2 ± 1.2 | 8.9 ± 1.6 |
| DMF | 20 ± 5 | 22 ± 9 | 12 ± 4 | 12 ± 3 |
| Rhodamine 123 | | | | |
| $IC_{50}$ (μg ml$^{-1}$) | 10.1 ± 2.4 | 1.2 ± 0.5 | 7.0 ± 3.6 | 9.8 ± 1.3 |
| RF | 21 ± 4 | 2.0 ± 0.6 | 8.7 ± 2.8 | 14 ± 7 |
| DMF | 18 ± 7 | 5.7 ± 3.8 | 3.3 ± 0.3 | 4.6 ± 0.8 |

Data shown are mean values ± s.d. from at least three experiments. [a]Resistance factor (RF) is expressed as the $IC_{50}$ of resistant cells divided by the $IC_{50}$ of the parental cells. [b]DMF (dose modifying factor) is expressed as the $IC_{50}$ without BSO treatment divided by the $IC_{50}$ in the presence of 25 μM BSO.

Figure 1 Effect of BSO on DNR (a) and Rh123 (b) accumulation in COR-L23 cells. COR-L23/P (circles) and COR-L23/R (triangles) were incubated in the presence of 25 μM BSO (closed symbols) or vehicle (open symbols) for 20–28 h. Cellular accumulation of 0.5 μM [$^3$H]DNR (data are means ± s.d. from three experiments, each performed in triplicate) and 0.01 μg ml$^{-1}$ Rh123 (a representative experiment out of three experiments is shown) was determined at the time points indicated.

Table II Effect of BSO on daunorubicin and rhodamine 123 accumulation

| | DNR (pmol per $10^6$ cells) | | Rh123 (relative to fluorescence in parent cells) | |
|---|---|---|---|---|
| | Control | + BSO | Control | + BSO |
| COR-L23/P | 308 ± 74 | 350 ± 98[a] | 1 | 1.35 ± 0.15[a] |
| COR-L23/R | 104 ± 25 | 359 ± 79[a] | 0.059 ± 0.025 | 0.71 ± 0.13[a] |
| GLC4 | 123 ± 23 | 130 ± 22 | 1 | 1.10 ± 0.09 |
| GLC4/ADR | 22 ± 10 | 111 ± 36[a] | 0.39 ± 0.07 | 0.96 ± 0.07[a] |
| MOR/P | 159 ± 26 | 291 ± 93[a] | 1 | 1.41 ± 0.17[a] |
| MOR/R0.2 | 59 ± 30 | 202 ± 58[a] | 0.21 ± 0.02 | 0.87 ± 0.26[a] |
| MOR/R0.4 | 41 ± 15 | 98 ± 17[a] | 0.037 ± 0.007 | 0.59 ± 0.14[a] |
| H69/P[b] | 187; 237 | 174; 263 | ND | ND |
| H69/LX4 (P-gp MDR) | 50 ± 15 | 44 ± 13 | ND | ND |

Steady-state accumulation of 0.5 μM [$^3$H]DNR (2 h) and 0.01 μg ml$^{-1}$ Rh123 (20 h) was measured in BSO-treated cells (25 μM, 20 h) or under normal conditions. Results are expressed as means ± s.d. from at least three experiments. ND, not determined. [a]Drug accumulation in the presence of BSO *vs* control is significantly different, $P \leq 0.05$, Student's paired *t*-test. [b]Data from two experiments.

increase in steady-state accumulation of DNR and Rh123 was measured in the parental MOR/P and COR-L23/P cells. However, no effect of BSO was measured in the GLC4 and H69/P cells. In contrast to the MRP MDR cell lines, BSO had no effect on the decreased DNR accumulation in P-gp MDR cell line H69/LX4.

Figure 2 shows that the BSO-induced increase in DNR and Rh123 accumulation in the COR-L23/R cells was caused by inhibition of the enhanced drug efflux. BSO did not affect the efflux of DNR and Rh123 in the parental COR-L23/P cells. Inhibition of the enhanced drug efflux was also observed in the other MRP MDR cell lines (data not shown).

### *Intracellular glutathione levels*

Since BSO is an inhibitor of glutathione synthesis and might affect drug transport via this pathway, we determined the effects of BSO treatment on cellular GSH content (Table III). Basal GSH levels did not correlate with resistance: the COR-L23/R cells had a lower, the GLC4/ADR cells a higher and the MOR/R cells a similar GSH content compared with their parental cell lines. These results are in agreement with reported data for these cell lines (Meijer *et al*., 1991; Rhodes and Twentyman, 1992). Incubation with 25 µM BSO for 20 h resulted in a similar percentage of GSH depletion in the resistant and parental cells (Table III).

### *Correlation between cellular GSH content and drug transport*

To evaluate the effect of GSH depletion by BSO on drug transport, we studied the concentration- and time-dependent

**Figure 2** Efflux of DNR (**a**) and Rh123 (**b**) in the presence of BSO in COR-L23 cells. COR-L23/P (circles) and COR-L23/R (triangles) cells were exposed to 25 µM BSO or vehicle for 20 h. Cells were preloaded with 0.5 µM [³H]DNR or 0.1 µg ml$^{-1}$ Rh123 for 60 min in the presence (closed symbols) or absence of BSO (open symbols). Retention of DNR and Rh123 was measured after suspending the cells in drug-free medium (open symbols) or in drug-free medium with 25 µM BSO (closed symbols). Data are means ± s.d. of three experiments.

**Figure 3** Dose–response curve of BSO in COR-L23/R and GLC4/ADR cells. COR-L23/R (open circles) and GLC4/ADR cells (closed circles) were incubated with various BSO concentrations for 20 h and then examined for (**a**) cellular GSH content and (**b**) DNR accumulation. Results are means ± s.d. of at least three experiments.

**Table III** Cellular GSH depletion by BSO

| | *Control (nmol per 10⁶ cells)* | *25 µM BSO (nmol per 10⁶ cells)* | *25 µM BSO Percentage of control* |
|---|---|---|---|
| COR-L23/P | 19.8 ± 3.0 | 8.1 ± 2.1 | 39 ± 12 |
| COR-L23/R | 13.5 ± 2.8[a] | 4.1 ± 1.1 | 31 ± 12 |
| GLC4 | 5.0 ± 0.7 | 1.3 ± 0.2 | 28 ± 5 |
| GLC4/ADR | 11.5 ± 2.3[a] | 2.3 ± 0.9 | 18 ± 10 |
| MOR/P | 22.3 ± 7.5 | 5.3 ± 3.2 | 22 ± 10 |
| MOR/R0.2 | 25.1 ± 6.6 | 4.3 ± 1.5 | 16 ± 3 |
| MOR/R0.4 | 19.5 ± 4.0 | 3.0 ± 0.8 | 16 ± 5 |

Cellular GSH content was determined with Ellman's substrate in cells treated with or without 25 µM BSO for 20 h. Results are expressed as means ± s.d. from 5–9 experiments, each performed in triplicate. [a]GSH content in resistant cells is significantly different ($P < 0.01$) from that in parental cells (Student's *t*-test).

effects of BSO in the resistant COR-L23/R and GLC4/ADR cells. A dose–response curve for BSO on GSH depletion and DNR accumulation is shown in Figure 3. Maximal effects on GSH depletion and DNR accumulation were measured in the COR-L23/R and GLC4/ADR cells at 25 µM and 100 µM BSO respectively. Intermediate levels of GSH depletion, caused by incubation with 2.5 µM BSO, resulted in only partial restoration of the DNR accumulation deficit. Incubation of the cells with DNR did not affect the cellular GSH content (data not shown).

Intermediate levels of GSH depletion could also be obtained by incubation of the resistant cells for shorter time periods with 25 µM BSO (Figure 4). A gradual decrease in cellular GSH content up to 50% was measured in the resistant COR-L23/R cells during a 4 h exposure to BSO (Figure 4a). A similar time course effect was measured for the increase in DNR accumulation (Figure 4b). In contrast to the rapid fall in the resistant cells, GSH levels were hardly affected during a 4 h incubation with BSO in the parental COR-L23/P cells. Prolongation of the incubation time with BSO resulted in similar GSH levels in sensitive and resistant cells.

The time- and concentration-dependent effects of BSO suggest that the effect of BSO on drug transport in MRP overexpression cells is caused by cellular GSH depletion. To exclude further a direct effect of BSO on drug transport, we increased cellular GSH content in BSO-treated cells with GSH ethyl ester (Table IV). In contrast to GSH itself, monoesters of GSH are well transported into cells and hydrolysed intracellularly to GSH (Meister, 1988). Monoesters of GSH have, therefore, been shown effective in increasing intracellular GSH levels even in the presence of BSO (Meister, 1988). As is shown in Table IV, incubation of the cells with 5 mM GSH ethyl ester indeed resulted in elevated cellular GSH levels. In the BSO-treated resistant cells, an increase in cellular GSH content was accompanied by a decrease in the DNR accumulation. No effect of GSH ethyl ester was measured on DNR accumulation in BSO-treated parental cells. This result proves that the effect of BSO on drug transport in MRP MDR cells involves cellular GSH depletion.

**Figure 4** Time-dependent effect of BSO in COR-L23 cells. COR-L23/P (open circles) and COR-L23/R cells (closed circles) were incubated for various time periods with 25 µM BSO and sampled for (**a**) cellular GSH content and (**b**) DNR acumulation (0.5 µM, added during the last 2 h of BSO treatment).

### *GSH transport*

To examine a further interaction between GSH and DNR transport, we measured the effects of DNR on the release of GSH into the medium. Since GSH that is released from cells

**Table V** Effect of DNR on GSH release into the medium

| Cell line | *GSH release (nmol per $10^6$ cells) after 60 min* Control | 25 µM DNR |
|---|---|---|
| COR-L23/P | 7.6 ± 1.4 (3) | 6.4 ± 1.1 (3) |
| COR-L23/R | 6.1 ± 1.8 (3) | 5.3 ± 1.8 (3) |
| GLC4 | 1.7 ± 0.5 (2) | 1.4 ± 0.3 (2) |
| GLC4/ADR | 5.3 ± 1.5 (3) | 5.3 ± 1.4 (3) |
| MOR/P | 4.8 ± 2.4 (4) | 3.8 ± 2.1 (3) |
| MOR/R0.2 | 8.0 ± 3.1 (4) | 7.5 ± 3.7 (3) |
| MOR/R0.4 | 11.2 ± 3.0 (5) | 10.7 ± 4.1 (4) |

GSH release was measured in the medium with Ellman's substrate following incubation of the cells with or without 25 µM DNR. Data are mean ± s.d. (number of experiments in parentheses, each performed in triplicate).

**Table IV** Modification of BSO effects by GSH ethyl ester

| | *Control* | | *2.5 µM BSO* | | *25 µM BSO* | |
|---|---|---|---|---|---|---|
| | *– ester* | *+ ester* | *– ester* | *+ ester* | *– ester* | *+ ester* |
| *GSH (nmol per $10^6$ cells)* | | | | | | |
| COR-L23/P | 19.9 ± 2.4 (3) | 26.8 ± 0.9 (3)[a] | | | 6.5 ± 0.5 (3) | 14.8 ± 2.6 (3)[a] |
| COR-L23/R | 15.9 ± 0.6 (3) | 25.6 ± 4.1 (3)[a] | 10.1 ± 1.0 (2) | 17.1 ± 0.2 (2) | 5.1 ± 1.3 (3) | 11.7 ± 0.8 (3)[a] |
| GLC4 | 5.4 ± 0.1 (2) | 8.1 ± 1.0 (2) | | | 1.5 ± 0.3 (2) | 4.2 ± 0.3 (2) |
| GLC4/ADR | 14.3 ± 2.1 (4) | 17.0 ± 0.7 (4) | 4.9 ± 0.1 (2) | 9.5 ± 2.0 (2) | 2.0 ± 0.5 (4) | 3.9 ± 0.7 (4)[a] |
| *Daunorubicin (pmol per $10^6$ cells)* | | | | | | |
| COR-L23/P | 350 ± 56 (3) | 305 ± 21 (3) | | | 422 ± 77 (3) | 427 ± 79 (3) |
| COR-L23/R | 112 ± 27 (3) | 66 ± 12 (3) | 213 ± 6 (2) | 158 ± 21 (2) | 331 ± 48 (3) | 260 ± 52 (3)[a] |
| GLC4 | 111 ± 10 (2) | 124 ± 8 (2) | | | 126 ± 7 (2) | 130 ± 9 (2) |
| GLC4/ADR | 12 ± 4 (4) | 11 ± 5 (4) | 31 ± 4 (2) | 14 ± 1 (2) | 90 ± 26 (4) | 54 ± 17 (4)[a] |

Cells were incubated with 0, 2.5 or 25 µM BSO for 20 h. Following co-incubation with 5 mM GSH ethyl ester for the last 4 h of the incubation, cellular GSH and DNR content was measured. Data shown are mean ± s.d. of 2–4 experiments (number in parentheses), each performed in triplicate. [a]Value (+ ester) significantly different from value (– ester), $P < 0.05$, Student's $t$-test.

can be degraded by γ-glutamyltranspeptidase, 0.2 mM acivicin, an inhibitor, was added to prevent degradation of GSH (Sze et al., 1993). Release of GSH was similar from COR-L23 parental and resistant cells, whereas it was increased in the resistant MOR/R and GLC4/ADR cells compared with their parental cells (Table V). If the transport of DNR were to interact with the transport of GSH, the effects would be maximal under conditions in which the active DNR transport is saturated. Previously, we have shown in the GLC4/ADR cells that the transport of DNR became saturated at concentrations (in the medium) higher than 5 μM and was almost completely saturated at 25 μM (Versantvoort et al., 1994). However, 25 μM DNR had no effect on GSH release in any of the cell lines (Table V).

*MRP RNA and protein expression*

It has been reported that oxidative stress, for example caused by cellular GSH depletion, increases expression of heat shock proteins, GSTs and other xenobiotic-metabolising enzymes (Bergelson et al., 1994; Sierra-Rivera et al., 1994). We examined whether cellular GSH depletion, due to BSO treatment, reduced the expression of MRP RNA and protein and reversed in this way the accumulation deficit. No effect of BSO was seen on RNA expression as measured by RT–PCR, or on protein expression by Western blotting with the MRPrl antibody (Figure 5).

### Discussion

The present study establishes that drug transport in MRP-overexpressing MDR tumour cells can be regulated by intracellular GSH levels. Depletion of cellular GSH levels in three MRP-mediated MDR human lung tumour cell lines, following exposure of the cells to BSO, results in an increase in drug accumulation associated with a potentiation of the drug toxicity. In an abstract by Longhurst et al. (1994), similar effects of BSO were found for an MRP-overexpressing, epirubicin-selected CCRF-CEM human leukaemia cell line. Further, we have shown that the expression of MRP is not affected by GSH depletion (Figure 5) and that restoration of cellular GSH levels in BSO-treated MRP-overexpressing cells reinduces the accumulation deficit of DNR (Table IV). In contrast to these effects on MRP MDR cell lines, cellular GSH depletion had no effect on the decreased DNR accumulation in the P-gp MDR H69/LX4 cell line (Table II). Thus, these data indicate that drug transport is regulated differently in MRP- and P-gp-overexpressing cells.

Transfection of the *MRP* gene in drug-sensitive cells has now been shown to confer MDR associated with a decreased accumulatior of drugs (Grant et al., 1994; Zaman et al., 1994). It is therefore likely that MRP is the drug transporter which has been shown functionally present in many non-P-gp-MDR cells (Slovak et al., 1988; McGrath et al., 1989; Kuiper et al., 1990; Coley et al., 1991). The pharmacological properties of drug transport in MRP- and P-gp-overexpressing cell lines show very similar characteristics (Broxterman and Versantvoort, 1995). Most of the drugs that are transported by P-gp (for example doxorubicin, DNR, VCR, VP-16 and Rh123), have now been reported to be transported in MRP-overexpressing cell lines (Slovak et al., 1988; Versantvoort et al., 1992, 1993; Twentyman et al., 1994). The accumulation of drugs is decreased due to an enhanced, energy-dependent efflux from the cells, and we have demonstrated that the transport of DNR in the MRP-overexpressing GLC4/ADR cells is saturable (Versantvoort et al., 1994) with an apparent $K_m$ of 1.4 μM which is similar to the $K_m$ of DNR transport found in P-gp MDR cell lines (Spoelstra et al., 1992). Nevertheless, there are important differences between MRP and P-gp in the modulation of the drug transport by resistance modifiers. Cyclosporin A and PSC-833, effective resistance-modifying agents for P-gp-mediated MDR, were shown to be less potent in reversing resistance in

**Figure 5** RNA and protein expression of MRP. (**a**) MRP RNA expression was determined by RT–PCR as described in the Materials and methods section. (**b**) Expression of the 190 kDa product of the *MRP* gene was measured by Western blotting with monoclonal antibody MRPrl.

the COR-L23/R cells (Barrand et al., 1993). In addition, Zaman et al. (1994) reported that cyclosporin A could not reverse the DNR accumulation deficit in the *MRP*-transfected cells. In contrast, we have shown that the isoflavonoid genistein increases DNR accumulation in several non-P-gp MDR cells but not in P-gp MDR cells (Versantvoort et al., 1993). It was shown in the GLC4/ADR cells that genistein inhibited the DNR transport by competition with DNR (Versantvoort et al., 1994), again indicating a different handling of the drugs by MRP and P-gp.

Interestingly, Jedlitschky et al. (1994) and Müller et al. (1994) have found that transport of leukotriene $C_4$ ($LTC_4$) and S-dinitrophenylglutathione (DNP-GS, glutathione S-conjugates) is increased in membrane vesicles from MRP-overexpressing cells as compared with the parental cells. Photoaffinity studies with $[^3H]LTC_4$ labelled the 190 kDa protein, proving that $LTC_4$ is transported by MRP (Jedlitschky et al., 1994). Thus, a physiological function of MRP might be the transport of organic anions such as the leukotrienes conjugated to GSH or cysteine (Leier et al., 1994).

The finding that MRP transports anionic glutathione S-conjugates and the present results that cellular GSH levels regulate drug transport activity have important implications for our understanding of the mechanism by which cytotoxic drugs are transported in MRP-overexpressing cells. The most obvious explanation is that MRP transports only negatively charged molecules. In that case, the hydrophobic, mostly cationic cytotoxic drugs involved in MDR, such as doxorubicin, DNR and VCR, should first be metabolised to become negatively charged, for example by conjugation with GSH, glucuronate or sulphate groups. This drug-conjugate might then be a substrate for MRP. The finding presented in this paper that cellular GSH depletion, following BSO treatment, inhibits the enhanced drug efflux (Figure 2) would then suggest that the drugs are transported in the form of a glutathione S-conjugate.

Although formation of conjugates with GSH has been implicated in drug resistance to alkylating agents such as melphalan and nitrogen mustard, there is no substantial evidence for conjugation of GSH with any of the MDR drugs (reviewed in Tew, 1994). After incubation of several drug-sensitive cells with DNR, no conjugates of DNR could be detected (de Jong et al., 1992). The lack of evidence for existence of glutathione S-conjugates with MDR drugs might be explained by a possible instability of the conjugate. In that case the conjugate of drugs with glutathione would be, as soon as it is formed, successively transported out of the cells and split in the extracellular medium into the single components GSH and cytotoxic drug. Transport of drugs out of MRP MDR cells would then give a rise in extracellular GSH levels. We have previously measured in GLC4/ADR cells that, at a DNR concentration of 25 $\mu$M, about 150 pmol DNR per $10^6$ cells $min^{-1}$ is transported (Versantvoort et al., 1994). If we assume a stoichiometry of one GSH molecule per molecule DNR transported, incubation of the resistant cells for one hour with 25 $\mu$M DNR should then increase the GSH release into the medium with 9 nmol GSH per $10^6$ cells $h^{-1}$. However, no increase in GSH release was measured upon DNR exposure in any of the cell lines (Table V). These results do not support a model in which drugs are transported out of MRP MDR cells in the form of glutathione S-conjugates. Furthermore, MRP-overexpressing cells are not cross-resistant to alkylating agents (Zijlstra et al., 1987; Rhodes and Twentyman, 1992), but have a similar cross-resistance spectrum as P-gp MDR cells. It has been shown by drug transport studies in plasma membrane vesicles of P-gp MDR cells that cytotoxic drugs are transported in free unconjugated form by P-gp (Horio et al., 1988). These findings suggest that cytotoxic drugs are transported out of the MRP-overexpressing cells not in the form of a glutathione S-conjugate.

Therefore, we would like to interpret the apparent paradox that overexpression of MRP results in transport of negatively charged molecules ($LTC_4$, DNP-GS) as well as transport of neutral or positively charged drugs (VP-16 and DNR), which has been shown to depend on cellular GSH levels, in terms of the following two models: (1) MRP is a bifunctional transport protein for anionic glutathione S-conjugates and cytotoxic drugs or (2) MRP is a glutathione S-conjugate transporter and activates an endogenous latent drug transport protein. In the first model, the cytotoxic drugs might form a ternary complex with glutathione S-conjugates, which is then transported as the complex by MRP. Cellular GSH depletion would then result in less complex formation and would therefore increase the cellular accumulation of drugs. In this model, modulation of drug transport is not separable from modulation of transport of glutathione S-conjugates. Awasthi et al. (1994) showed that transport of doxorubicin and DNP-GS in membrane vesicles of erythrocytes could inhibit each other. It was suggested that a 38 kDa protein was involved in the transport. On the other hand, doxorubicin did not inhibit, and vincristine and vinblastine inhibited only at high concentrations (100 $\mu$M), the transport of LTC4 and DNP-GS by MRP (Müller et al., 1994). Thus, cytotoxic drugs do not stimulate the transport of glutathione S-conjugates in MRP-overexpressing cells. Rather than forming a complex with glutathione S-conjugates, transport of these compounds might induce a conformational change in MRP which enables the efflux of cytotoxic drugs. In that case modulation of the transport of glutathione S-conjugates, for example by GSH depletion, would affect the drug transport activity. The transport of drugs could additionally be inhibited with resistance modulators by competition for the drug binding site such as shown for genistein (Versantvoort et al., 1994).

In the second model, the cytotoxic drugs would be transported not by MRP but by a latent drug transport protein present in the cells. MRP might form a membrane-bound complex with the drug transport protein. The transport of glutathione S-conjugates would then allow the transport of drugs through a series of conformational changes. As in the above described model, inhibition of the glutathione S-conjugate transport would inhibit the drug transport, but drug transport could also be inhibited at its drug binding sites.

In conclusion, our results indicate that transport of drugs in MRP-overexpressing but not in P-gp-overexpressing MDR cells can be regulated by cellular GSH levels. In the models described above we have assumed that GSH depletion inhibited the drug transport through inhibition of the transport of glutathione S-conjugates by MRP. However, the possibility that GSH depletion causes a conformational change in MRP, which inhibits the transport of drugs, or activates an inhibitory molecule cannot be excluded. In this case MRP-mediated glutathione S-conjugate and drug transport can be completely distinct and separable. Further inhibition studies as well as studies on transport of cytotoxic agents in inside-out plasma membrane vesicles of MRP-overexpressing cells will provide evidence in which form the cytotoxic agents are transported and their dependence on GSH and/or glutathione S-conjugates.

**Abbreviations** MDR, multidrug resistance; P-gp, P-glycoprotein; MRP, multidrug resistance-associated protein; GSH, glutathione; GST, glutathione S-transferase; DNR, daunorubicin; VCR, vincristine; Rh123, rhodamine 123; BSO, DL-buthionine (S,R)-sulphoximine; $LTC_4$, leukotriene $C_4$; DNP-GS, S-dinitrophenylglutathione

**Acknowledgement**

HJB is supported by the Dutch Cancer Society Grant NKBVU 93-626.

**References**

AWASTHI S, SINGHAL SS, SRIVATAVA SK, ZIMNIAK P, BAJPAI KK, SAXENA M, SHARMA R, ZILLER III SA, FRENKEL EP, SINGH SV, HE NG AND AWASTHI YC. (1994). Adenosine triphosphate-dependent transport of doxorubicin, daunomycin, and vinblastine in human tissues by a mechanism distinct from the P-glycoprotein. J. Clin. Invest., 93, 958-965.

BARRAND MA, RHODES T, CENTER MS AND TWENTYMAN PR. (1993). Chemosensitisation and drug accumulation effects of cyclosporin A, PSC833 and verapamil in human MDR large cell lung cancer cells expressing a 190k membrane protein distinct from P-glycoprotein. Eur. J. Cancer, 29A, 408-415.

BARRAND MA, HEPPEL-PARTON AC, WRIGHT KA, RABBITS PH AND TWENTYMAN PR. (1994). A 190k protein overexpressed in non-P-glycoprotein containing MDR cells and its relation to the MRP gene. J. Natl Cancer Inst., 86, 110-117.

BERGELSON S, PINKUS R AND DANIEL V. (1994). Intracellular glutathione levels regulate Fos/Jun induction and activation of glutathione S-transferase gene expression. Cancer Res., 54, 36-40.

BROXTERMAN HJ AND VERSANTVOORT CHM. (1995). Pharmacology of drug transport in multidrug resistant tumor cells. In *Alternative Mechanisms of Multidrug Resistance in Cancer*, Kellen JA (ed), pp. 67-81. Birkäuser: Boston.

COLE SPC, DOWNES HF, MIRSKI SEL AND CLEMENTS DJ. (1990). Alterations in glutathione and glutathione-related enzymes in a multidrug-resistant small cell lung cancer cell line. *Mol. Pharmcol.*, **37**, 192-197.

COLE SPC, BHARDAWAJ G, GERLACH JH, MACKIE JE, GRANT CE, ALMQUIST KC, STEWART AJ, KURZ EU, DUNCAN AMV AND DEELEY, RG. (1992). Overexpression of a novel transporter gene in a multidrug resistant human lung cancer cell line. *Science*, **258**, 1650-1654.

COLEY HM, WORKMAN P AND TWENTYMAN PR. (1991). Retention of activity by selected anthracyclines in a multidrug resistant human large cell lung carcinoma line without P-glycoprotein hyperexpression. *Br. J. Cancer*, **63**, 351-357.

DE JONG S, ZIJLSTRA JG, DE VRIES EGE AND MULDER NM. (1990). Reduced DNA topoisomerase II activity and drug-induced DNA cleavable activity in an adriamycin-resistant human small cell lung carcinoma cell line. *Cancer Res.*, **50**, 304-309.

DE JONG J, KUIPER CM, BAST A, AND VAN DER VIJGH WJF. (1992). Interpretation of murine tumor pharmacokinetics of anthracyclines and metabolites through in vivo antitumor activities in three different cell lines. J. de Jong, Thesis, Free University Amsterdam.

DUSRE L, MIMNAUGH EG, MYERS CE AND SIHNA BK. (1989). Potentiation of doxorubicin cytotoxicity by buthionine sulfoximine in multidrug-resistant human breast tumor cells. *Cancer Res.*, **49**, 511-515.

FLENS MJ, IZQUIERDO MA, SCHEFFER GL, FRITZ JM, MEIJER CJLM, SCHEPER RJ AND ZAMAN GJR. (1994). Immunohistochemical detection of the multidrug resistance-associated protein MRP in human multidrug-resistant tumor cells by monoclonal antibodies. *Cancer Res.*, **54**, 4557-4563.

GOTTESMAN MM AND PASTAN I. (1993). Biochemistry of multidrug resistance mediated by the multidrug transporter. *Annu. Rev. Biochem.*, **62**, 385-427.

GRANT CE, VALDIMARSSON G, HIPFNER E, ALMQUIST KC, COLE SPC AND DEELEY RG. (1994). Overexpression of multidrug resistance-associated protein (MRP) increases resistance to natural product drugs. *Cancer Res.*, **54**, 357-361.

HORIO M, GOTTESMAN MM AND PASTAN I. (1988). ATP-dependent transport of vinblastine in vesicles from human multidrug-resistant cells. *Proc. Natl Acad. Sci. USA*, **85**, 3580-3584.

JEDLITSCHKY G, LEIER I, BUCHHOLZ U, CENTER MS AND KEPPLER D. (1994). ATP-dependent transport of glutathione *S*-conjugates by the multidrug resistance-associated protein. *Cancer Res.*, **54**, 4833-4836.

KRAMER RA, ZAKHER J AND KIM G. (1988). Role of the glutathione redox cycle in acquired and de novo multidrug resistance. *Science*, **241**, 694-696.

KRISHNAMACHARY N AND CENTER MS. (1993). The MRP gene associated with a non-P-glycoprotein multidrug resistance encodes a 190-kDa membrane bound glycoprotein. *Cancer Res.*, **53**, 3658-3661.

KUIPER CM, BROXTERMAN HJ, BAAS F, SCHUURHUIS GJ, HAISMA HJ, SCHEFFER GL, LANKELMA J AND PINEDO HM. (1990). Drug transport variants without P-glycoprotein overexpression from a human squamous lung cancer cell line after selection with doxorubicin. *J. Cell. Pharmacol.*, **1**, 35-41.

LARSSON R, BERGH J AND NYGREN P. (1991). Combination of cyclosporin A and buthionine sulfoximine (BSO) as a pharmacological strategy for circumvention of multidrug resistance in small cell lung cancer cell lines selected for resistance to doxorubicin. *Anticancer Res.*, **11**, 455-460.

LEIER I, JEDLITSCHKY G, BUCHHOLZ U AND KEPPLER D. (1994). Characterization of the ATP-dependent leukotriene C4 export carrier in mastocytoma cells. *Eur. J. Biochem.*, **220**, 599-606.

LONGHURST T, HARVIE RM, DAVEY MW AND DAVEY RA. (1994). Multidrug resistance (MDR) mechanisms in epirubicin selected CCRF-CEM human leukemia cells which overexpress MRP. *Proc. Am. Assoc. Cancer Res.*, **85**, 13 (73).

LUTZKY J, ASTOR MB, TAUB RN, BAKER MA, BHALLA K, GERVASONI JR JE, ROSADO M, STEWART V, KRISHNA S AND HINDENBURG AA. (1989). Role of glutathione and dependent enzymes in anthracycline-resistant HL60/AR cells. *Cancer Res.*, **49**, 4120-2125.

MCGRATH T, LATOUD C, ARNOLD ST, SAFA AR, FELSTED L AND CENTER MS. (1989). Mechanisms of multidrug resistance in HL60 cells: analysis of resistance associated membrane proteins and levels of *mdr* gene expression. *Biochem. Pharmacol.*, **38**, 3611-3619.

MEISTER A. (1988). Novel drugs that affect glutathione metabolism. In *Mechanisms of Drug Resistance in Neoplastic Cells*, Woolley PV and Tew KD. (eds) pp. 99-126. Academic Press and Harcourt Brance Javanovich: New York.

MEIJER C, MULDER NH, TIMMER-BOSSCHA H, PETERS WHM AND DE VRIES EGE. (1991). Combined *in vitro* modulation of adriamycin resistance. *Int. J. Cancer*, **49**, 582-586.

MÜLLER M, MEIJER C, ZAMAN GJR, BORST P, SCHEPER RJ, MULDER NH, DE VRIES EGE AND JANSEN PLM. (1994). Overexpression of the gene encoding the multidrug resistance-associated protein results in increased ATP-dependent glutathione S-conjugate transport. *Proc. Natl Acad. Sci. USA*, **91**, 13033-13027.

RHODES T AND TWENTYMAN PR. (1992). A study of ethacrynic acid as a potential modifier of melphalan and cisplatin sensitivity in human lung cancer parental and drug-resistant cell lines. *Br. J. Cancer*, **65**, 684-690.

SEDLAK J AND LINDSAY RH. (1968). Estimation of total, protein-bound, and nonprotein sulfhydryl groups in tissue with Ellman's reagent. *Anal. Biochem.*, **25**, 192-205.

SIERRA-RIVERA E, MEREDITH MJ, VOORHEES GJ, OBERELEY LW, EISERT DR AND FREEMAN ML. (1994). Synthesis of heat shock proteins following oxidative challenge: role of glutathione. *Int. J. Hyperthermia*, **10**, 573-586.

SLOVAK ML, HOELTGE GA, DALTON WS AND TRENT JM. (1988). Pharmacological and biological evidence for differing mechanisms of doxorubicin resistance in two human tumor cell lines. *Cancer Res.*, **48**, 2793-2797.

SLOVAK ML, HO J, DEELEY RG AND COLE SPC. (1993). Localisation of a novel multidrug resistance-associated gene in the HT1080/DR4 and H69/AR human tumor cell lines. *Cancer Res.*, **53**, 3221-3225.

SPOELSTRA EC, WESTERHOFF HV, DEKKER H AND LANKELMA J. (1992). Kinetics of daunorubicin transport by P-glycoprotein of intact cancer cells. *Eur. J. Biochem.*, **207**, 567-579.

SZE G, KAPLOWITZ N, OOKHTENS M AND SHELLY CL. (1993). Bidirectional membrane transport of intact glutathione in Hep G2 cells. *Am. J. Physiol.*, **265**, G1128-G1134.

TEW KD. (1994). Glutathione-associated enzymes in anticancer drug resistance. *Cancer Res.*, **54**, 4313-4320.

TWENTYMAN PR, FOX NE, WRIGHT KA AND BLEEHEN N. (1986). Derivation and preliminary characterisation of Adriamycin resistant lines of human lung cancer cells. *Br. J. Cancer*, **53**, 529-537.

TWENTYMAN PR, RHODES T AND RAYNER S. (1994). A comparison of rhodamine 123 accumulation and efflux in cells with P-glycoprotein-mediated and MRP-associated multidrug resistance phenotypes. *Eur. J. Cancer*, **30A**, 1360-1369.

VERSANTVOORT CHM, BROXTERMAN HJ, PINEDO HM, DE VRIES EGE, FELLER N, KUIPER CM AND LANKELMA J. (1992). Energy-dependent processes involved in reduced drug accumulation in multidrug-resistant human lung cancer cell lines without P-glycoprotein expression. *Cancer Res.*, **52**, 17-23.

VERSANTVOORT CHM, SCHUURHUIS GJ, PINEDO HM, EEKMAN CA, KUIPER CM, LANKELMA J AND BROXTERMAN HJ. (1993). Genistein modulates the decreased drug accumulation in non-P-glycoprotein mediated multidrug resistant tumour cells. *Br. J. Cancer*, **68**, 939-946.

VERSANTVOORT CHM, BROXTERMAN HJ, LANKELMA J, FELLER N AND PINEDO HM. (1994). Competitive inhibition by genistein and ATP dependence of daunorubicin transport in intact MRP overexpressing human small cell lung cancer cells. *Biochem. Pharmacol.*, **48**, 1129-1236.

VERSTANTWOORT CHM, WITHOFF S, BROXTERMAN HJ, KUIPER N, SCHEPER RJ AND DE VRIES EGE. (1995). Resistance-associated factors in human small cell lung carcinomas GLC4 sublines with increasing adriamycin resistance. *Int. J. Cancer*, **60** (in press).

ZAMAN GJR, VERSANTVOORT CHM, SMIT JJM, EIJDEMS EWHM, DE HAAS M, SMITH AJ, BROXTERMAN HJ, MULDER NH, DE VRIES EGE, BAAS F AND BORST P. (1993). Analysis of the expression of *MRP*, the gene for a new putative transmembrane drug transporter, in human multidrug resistant lung cancer cell lines. *Cancer Res.*, **53**, 1747-1750.

ZAMAN GJR, FLENS MJ, VAN LEUSDEN MR, DE HAAS M, MÜLDER HS, LANKELMA J, PINEDO HM, SCHEPER RJ, BROXTERMAN HJ AND BORST P. (1994). The human multidrug resistance-associated protein MRP is a plasma membrane drug-efflux pump. *Proc. Natl Acad. Sci. USA*, **91**, 8822-8826.

ZIJLSTRA JG, DE VRIES EGE AND MULDER NH. (1987). Multifactorial drug resistance in an Adriamycin-resistant human small cell lung carcinoma cell line. *Cancer Res.*, **47**, 1780-1784.